# Platelet activation in diabetic mice models: the role of vascular endothelial cell-derived protein disulfide isomerase-mediated GP IIb/IIIa receptor activation

**DOI:** 10.18632/aging.102192

**Published:** 2019-08-22

**Authors:** Ran-Ran Qin, Hui Zhu, Feng Wang, Ming Song, Pei-Lin Lin, Yan-Qiu Xing, Wei Zhang, Ming Zhong, Zhi-Hao Wang

**Affiliations:** 1 The Key Laboratory of Cardiovascular Remodeling and Function Research, Chinese Ministry of Education, Chinese National Health Commission and Chinese Academy of Medical Sciences, The State and Shandong Province Joint Key Laboratory of Translational Cardiovascular Medicine, Department of Cardiology, Qilu Hospital of Shandong University, Jinan, Shandong 250012, China; 2 Department of Cardiology, The Affiliated Cardiovascular Hospital of Qingdao University, Qingdao, Shandong 266071, China; 3 Department of Geriatric Medicine, Qilu Hospital of Shandong University, Key Laboratory of Cardiovascular Proteomics of Shandong Province, Qilu Hospital of Shandong University, Jinan, Shandong 250012, China

**Keywords:** diabetes mellitus, protein disulfide isomerase, platelet activation, endothelial microparticle, glycoprotein IIb/IIIa receptor

## Abstract

GP IIb/IIIa receptor activation plays an important role in thrombosis. The mechanism of early activation of GP IIb/IIIa receptors in diabetic conditions remains unknown. The purpose of this study was to investigate the release of Endothelial microparticle (EMP)-associated protein disulfide isomerase (PDI) after endothelial cell injury induced in diabetes and the changes in platelet activation. We produced an animal model of type 2 diabetes mellitus using ApoE^−/−^ mice. Normal ApoE^−/−^ and diabetic mice were allocated to four groups (n = 15): normal diet, normal diet plus rutin, diabetic, and diabetes plus rutin. The EMP-PDI content and GP IIb/IIIa expression of mice platelets were determined. In addition, EMPs obtained from the four groups were pretreated with the PDI inhibitor rutin; then, their effects on the platelets of normal C57 mice were characterized. Compared with the normal diet group, the diabetic group had significantly increased plasma EMP-PDI content and accelerated platelet activation by increased GP IIb/IIIa expression. In conclusion, EMP-PDI promotes early platelet activation through glycoprotein (GP) IIb/IIIa receptors present on platelet surface in the diabetic state. However, this process could be partially suppressed by the administration of rutin.

## INTRODUCTION

The prevalence of type 2 diabetes mellitus is rapidly increasing, and it has emerged as a major public health problem worldwide. One of the most important complications of diabetes mellitus is cardiovascular disease [1–3]. Because most acute cardiovascular events are associated with thrombosis, intensive antiplatelet therapy offers a major clinical benefit to diabetic patients. However, diabetic patients have significantly higher platelet aggregation and activation than non-diabetic patients, including those undergoing dual antiplatelet therapy [4–6]. This suggests that the mechanism of platelet activation in the diabetic state has not been fully elucidated.

In thrombosis, platelet activation serves as the initiation factor. However, the ultimate common pathway of platelet aggregation is the activation of glycoprotein (GP) IIb/IIIa receptors present on platelet surface [7–9]. The identification of the key molecules that accurately regulate the activation of these receptors may provide a novel approach for antiplatelet therapy.

Under physiological conditions, the GP IIb/IIIa receptors are present in a low-affinity state and cannot bind to fibrinogen. However, following conformational changes, these receptors are transformed into a high-affinity state wherein they can bind to fibrinogen and promote thrombosis. This process relies on the destruction of disulfide bonds present on these receptors, such as Cys5–Cys435, Cys663–Cys687, and a third Cys-rich repetitive zone, to be transformed into the ligand-binding, high-affinity state and evoke conformational changes in GP IIb/IIIa receptors [10]. Protein disulfide isomerase (PDI) is an oxidation–reduction enzyme responsible for catalyzing disulfide bond formation [11–13]. It can directly act on GP IIb/IIIa receptors to promote conformational changes by regulating disulfide bond isomerization [10, 14], which in turn accelerates thrombosis. Therefore, PDI is the key enzyme regulating the activation of GP IIb/IIIa receptors. Studies have suggested that PDI is involved in the early activation of platelets [15], but its origin and mechanism of action in the diabetic state remains unknown.

Previous studies have found that there is an increase in the level of microparticles (MPs) in the culprit artery in acute myocardial infarction, which is related to thrombosis [16]. MPs are shed virtually by all cell types during cell growth, proliferation, and apoptosis, which increase under pathological conditions [17–21]. Platelet-derived MPs (PMPs) have been shown to carry PDI [22, 23], which can directly promote conformational changes in GP IIb/IIIa receptors, thereby activating platelets [24]. The coagulation-promoting effect of PMP is 50–100 times higher than that of activated platelets [22, 23]. However, Kim et al. [25] have reported that platelet-derived PDI only increased circulating PDI level, which had a cascade amplification effect on platelet activation. Normal endothelial cells have anticoagulant effects, but most of them also release large amounts of MPs when their function is impaired in metabolic syndrome and diabetes [21]. Therefore, it is unknown whether the endothelial microparticles activate platelets by carrying PDI or they are early promoters of platelet activation in the diabetic state.

In the present study, we aimed to test the hypothesis that the levels of PDI-carrying EMPs drastically increase in the diabetic state after endothelial cell injury and that PDI is the key factor that promotes early platelet activation in the diabetic state. To this end, we determined the changes in EMP level, EMP-PDI release, and platelet activity in the plasma of diabetic mice. In addition, we used PDI inhibitors to inhibit EMP-PDI function to observe the changes in platelet activation. Using this, we intend to identify a novel therapeutic target for platelet inhibition.

## RESULTS

### Establishment of animal models of type 2 diabetes using ApoE^-/-^ mice

**Figure 1:**
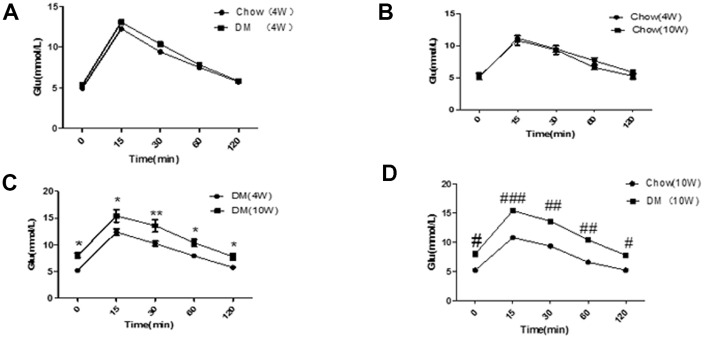
**Results of the glucose tolerance test in ApoE^-/-^ mice.** (**A**) Comparison of IPGTT in two groups of mice at four weeks; (**B**) Comparison of IPGTT before and after feeding in the normal diet group; (**C**) Comparison of IPGTT before and after feeding in the diabetic group. *P < 0.05, **P < 0.01, DM (10W) versus DM *(4W)*; (**D**) The IPGTT results were compared between the two groups at 10 weeks. *^#^P < 0.05, ^##^P < 0.01, ^###^P < 0.001, Chow (10W) versus DM (10W). Chow, normal diet group; DM, diabetic group* (n = 10–30). Data were analyzed using one-way ANOVA.

**Figure 2:**
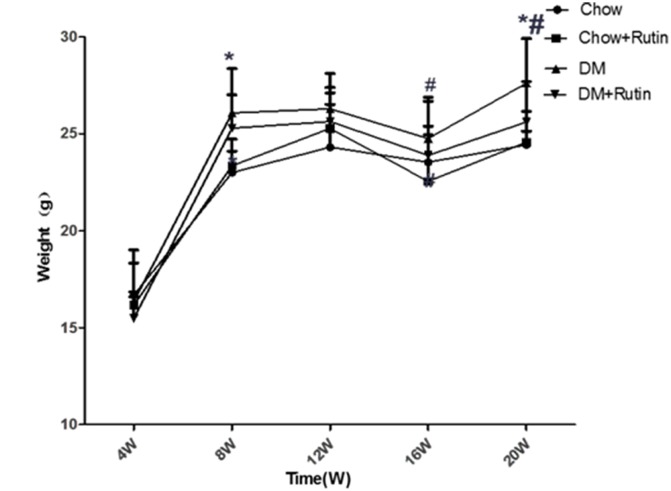
**ApoE^-/-^ body weight variation**. Measurement of the body weight of mice, *P < 0.05, versus Chow, ^#^*P* < 0.05, versus Chow + Rutin. *Chow, normal group; Chow + Rutin, normal diet plus rutin; DM, Diabetic group; DM + Rutin, diabetic group plus rutin* (n = 4–19). Data were analyzed using one-way ANOVA.

The IPGTT result did not show significant differences between mice in the normal diet group and those in the diabetes group at week 4 (*P* < 0.05, Figure 1A). In addition, there was no significant difference among mice of different ages in the normal diet group (*P* < 0.05, Figure 1B); At week 10, the IPGTT result in mice with diabetes was higher than that reported at week 4 in mice with diabetes and mice receiving a normal diet. The differences were statistically significant (*P* < 0.05, Figure 1C and 1D, respectively). At week 4, no significant difference was observed in the weight of male mice between the two groups (*P* > 0.05). At week 12, no significant difference was observed in the weight of mice between the diabetes group and the normal diet group (*P* > 0.05, Figure 2). These results indicated that the animal model of ApoE^-/-^ mice with type 2 diabetes was successfully established.

### FCM was used to detect the CD144-positive EMP level in the plasma of mice

**Figure 3:**
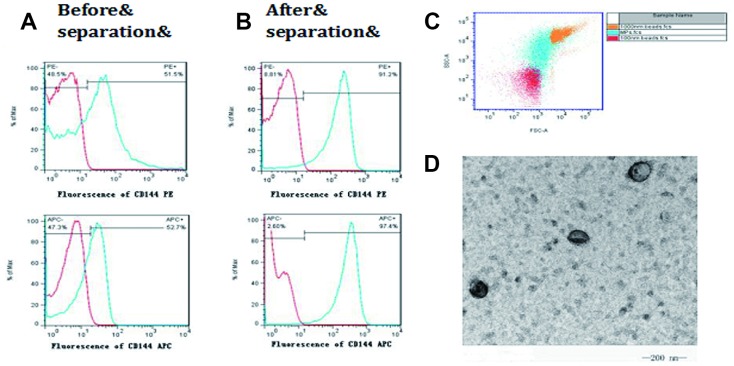
**Isolation and identification of microparticles from endothelial cells.** CD144 was used to mark endothelial cell-derived microparticles (EMP). (**A**) The positive rate of CD144 in the plasma before isolation; (**B**) The positive rate of CD144 in the plasma after isolation; (**C**) The isolated microparticles were further selected, using FCM, with the standards of 100 nm and 1000 nm; (**D**) Transmission electron microscopy was used to observe the obtained microparticles with a diameter >100 nm.

CD144 was used to mark EMP. After treatment with the magnetic cell separation technique (MACS) and ultracentrifugation, the positive rate of CD144-positive EMP increased from 50% to 90% (Figures 3A and 3B). The separated microparticles were further selected using FCM with the standard of 100 nm and 1000 nm fluorescent microsphere. (Figure 3C). Transmission electron microscopy was used to observe the obtained microparticles, which were typical double-membrane follicles with a diameter >100 nm (Figure 3D).

### The content of CD144-positive EMPs in the plasma of diabetic mice was increased, and PDI was presented on EMP. The content of EMP-PDI was significantly increased in the diabetic group

**Figure 4:**
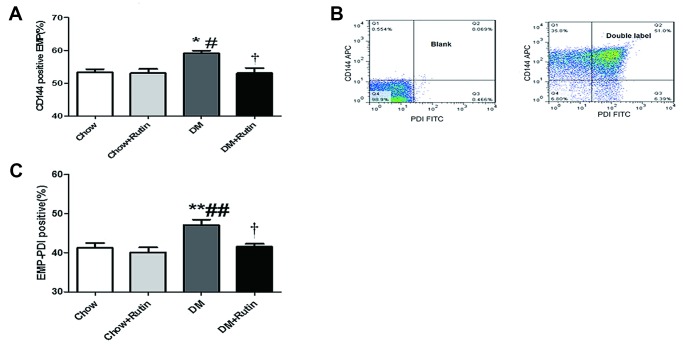
**PDI was carried by EMPs, and the contents of CD144-positive EMPs and EMP-PDI in the plasma were increased in the diabetic group.** (**A**) The content of CD144-positive EMPs in the plasma of diabetic mice was increased; (**B**) Dual-color flow cytometry was used to show that PDI is carried by the particles in endothelial cells; (**C**) The content of EMP-PDI in the plasma of diabetic mice was increased;*P < 0.05, **P < 0.01, ***P < 0.001 versus Chow; ^#^P < 0.05, ^##^P < 0.01, ^###^P < 0.001 versus Chow + Rutin; ^†^P < 0.05, ^††^P < 0.01, ^‡^P < 0.001 versus DM. Chow, normal group; Chow + Rutin, normal diet plus rutin; DM, Diabetic group; DM + Rutin, diabetic group plus rutin (n = 5–11). Data were analyzed using one-way ANOVA.

FCM was used to detect the CD144-positive EMP level in the plasma of mice. The results indicated that, compared to the normal diet group, the CD144-positive EMP levels in mice in the diabetes group were increased, and the difference was statistically significant (*P* < 0.05, Figure 4A). Compared to the diabetes group, the levels of CD144-positive EMPs in mice in the diabetes plus rutin group was significantly decreased (*P* < 0.05). This result indicated the presence of obvious endothelial injury in the blood vessels of diabetic mice, more EMPs were released from damaged endothelial cells and rutin may reduce the release of EMPs in diabetic state.

CD144-APC and PDI-FITC dual-color FCM confirmed that PDI was presented on EMP (Figure 4B). The EMP-PDI levels in mice in the diabetes group was higher than that observed in other groups, and the difference was statistically significant (*P* < 0.05~0.01, Figure 4C).

### Type 2 diabetic ApoE^-/-^ mice showed hypercoagulability, increased platelet activation, and increased PDI content, which was reversed by rutin intervention

**Figure 5:**
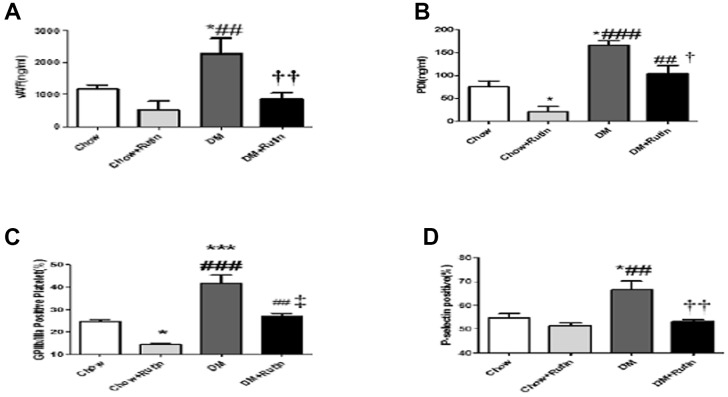
**The plasma content of VWF and PDI was detected and the activity of platelet between the four groups was compared.** (**A**) The VWF content in the plasma was detected using ELISA; (**B**) The PDI content in the plasma was detected using ELISA; (**C**) Flow cytometry was used to detect activated GP IIb/IIIa on the platelet surface; (**D**) Flow cytometry was used to detect P-selectin expression on the platelet surface *P < 0.05, **P < 0.01, ***P < 0.001, versus Chow; ^#^P < 0.05, ^##^P < 0.01, ^###^P < 0.001, versus Chow + Rutin, ^†^P < 0.05, ^††^P < 0.01, ^‡^P < 0.001, versus DM. Chow, normal group; Chow + Rutin, normal diet plus rutin; DM, Diabetic group; DM + Rutin, diabetic group plus rutin (n = 3–8). Data were analyzed using one-way ANOVA.

ELISA was used to detect the expression level of VWF and PDI in the plasma of mice. The results indicated that VWF and PDI levels in mice in the diabetes group were higher than those observed in the normal diet group, with the difference being statistically significant (*P* < 0.05). PDI content in mice fed with a normal diet plus rutin were significantly reduced compared to the normal diet group, and the rutin-fed mice with diabetes mellitus showed a marked decrease in PDI and VWF levels compared to the diabetes mellitus group (*P* < 0.01 and *P* < 0.05 respectively, Figure 5A and 5B).

### FCM was used to detect activated GP IIb/IIIa and P-selectin expression levels on the platelet surface of mice in each group

The results indicated activated GP IIb/IIIa and P-selectin expression in the diabetes group increased significantly compared to those observed in the normal diet group (*P* < 0.001 and *P* < 0.05 respectively, Figure 5C and 5D). However, activated GP IIb/IIIa and P-selectin expression levels in the diabetes plus rutin group were significantly lower than in the diabetes group. This indicates that rutin can block activation of GP IIb/IIIa receptors and P-selectin expression in diabetic mice.

### EMP-mediated PDI-dependent platelet activation, while RL90 and rutin inhibit this process

**Figure 6:**
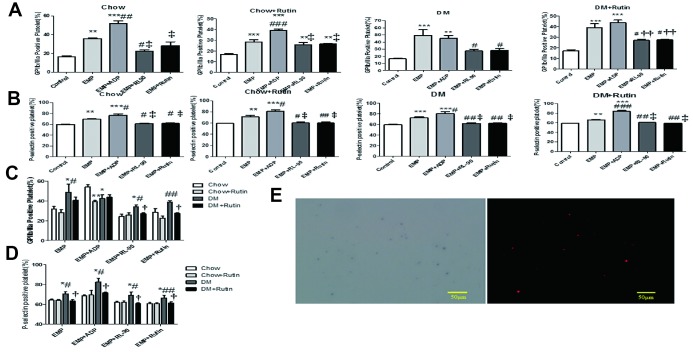
**Platelets of normal C57 mice were stimulated with EMP and pretreatment EMPs.** Adenosine diphosphate (ADP) (10 μg/mL), RL90 (1 μg/mL), and rutin (60 μM) were used to pretreat EMPs obtained from four groups; then the EMP and the pretreatment EMP were used to stimulate the platelets of normal C57 mice, respectively. FCM was used to detect the level of platelet activation. EMP from the diabetic mice can significantly activate platelets, while RL90 and rutin could inhibit this process. Data were analyzed using one-way ANOVA. (**A**) Comparisons of the GP IIb/IIIa receptor expression level on the platelet surface of C57 mice stimulated using different pretreated EMPs, within each group. The GP IIb/IIIa receptor expression significantly increased in the EMP stimulus subgroup. ^*^P < 0.05, ^**^P < 0.01, ^***^P < 0.001 versus Control; ^#^P < 0.05, ^##^P < 0.01, ^###^P < 0.001 versus EMP; P < 0.05, ^††^P < 0.01, ^‡^P < 0.001 versus EMP + ADP (n = 3–5). (**B**) Comparisons of the expression level of P-selectin on the platelet surface of C57 mice stimulated using different pretreated EMPs, within each group. The P-selectin expression was significantly increased in the EMP stimulus subgroup. ^*^P < 0.05, ^**^P < 0.01, ^***^P < 0.001 versus Control; ^#^P < 0.05, ^##^P < 0.01, ^###^P < 0.001 versus EMP; ^†^P < 0.05, ^††^P < 0.01, ^‡^P < 0.001 versus EMP + ADP (n = 3–10). (**C**) Comparisons of the GP IIb/IIIa receptor expression level on the platelet surface of normal C57 mice among the four groups stimulated using different pretreated EMP. ^*^P < 0.05, ^**^P < 0.01,^***^P < 0.001 versus Chow; ^#^P < 0.05, ^##^P < 0.01, ^###^P < 0.001 versus Chow + Rutin; ^†^P < 0.05, ^††^P < 0.01, ^‡^ P < 0.001 versus DM (n = 3–8). (**D**) Comparisons of the P-selectin expression level on the platelet surface of normal C57 mice among the four groups stimulated using different pretreated EMP. ^*^P < 0.05, ^**^P < 0.01, ^***^P < 0.001 versus Chow; ^#^P < 0.05, ^##^P < 0.01, ^###^P < 0.001 versus Chow + Rutin; ^†^P < 0.05, ^††^P < 0.01, ^‡^ P < 0.001 versus DM (n = 3–9). (**E**) The Duolink® *in-situ* proximity ligation assay was used to detect the GPIIb/IIIa receptor and EMP-PDI on the platelet surface. The red dot granule represents GPIIb/IIIa receptor binding to PDI (scale = 50 μm).

EMPs derived from the four groups (normal diet, normal diet plus rutin, diabetic, and diabetic plus rutin groups) were respectively pretreated with ADP (10 μg/mL), RL90 (1 μg/mL) and rutin (60 μM). Subsequently, the EMPs and the pretreatment EMP were used to stimulate the platelets in normal C57 mice. FCM was used to determine the platelet activation level. The results indicated that activated GP IIb/IIIa and P-selectin expression levels stimulated by EMPs obtained from four groups were significantly higher compared to those observed in the blank control group. Notably, the activated GP IIb/IIIa and P-selectin expression levels on the platelet surface, stimulated by EMP incubated with RL90 or rutin, were lower than those reported in the EMP stimulus group (*P* < 0.05~0.001, Figure 6A and 6B). These results indicate that both in normal diet group and in diabetes group, EMP promotes platelet activation, while PDI inhibitors such as RL90 and rutin inhibit EMP-mediated PDI-dependent platelet activation.

### EMPs in diabetic mice significantly activated platelets, PDI inhibitors RL90 and rutin inhibited the process

The EMP obtained from different groups and the pretreatment EMP (incubated by ADP, RL90, and rutin) were used to stimulate platelets in normal C57 mice. The activated GP IIb/IIIa and P-selectin expression levels on the platelet surface were compared among the four groups under the same stimulation condition. The results showed that, under the EMP stimulus, the GP IIb/IIIa and P-selectin expression levels on the platelet surface in the diabetes group were significantly higher than those in the normal diet group *(P* < 0.05~0.001, Figure 6C and 6D).

In EMP stimulation through pretreatment with ADP, the GP IIb/IIIa expression levels on the platelet surface in the diabetes group were statistically significantly reduced compared to the normal diet group (*P <* 0.05, Figure 6C). This finding indicates that treatment with ADP was not the main mode for platelet activation in diabetic mice.

In EMP stimulation through pretreatment with RL90 or rutin, the GP IIb/IIIa and P-selectin expression levels on the platelet surface in the diabetes plus rutin group were statistically significantly reduced compared to the diabetes group (*P* < 0.05, Figure 6C and 6D). The result shows that RL90 and rutin may partially inhibit the PDI pathway under diabetic states and attenuate the platelet activation.

### Duolink® *in-situ* PLA confirmed the interaction between the PDI on EMP and the GP IIb/IIIa receptor on the platelet surface

The results showed that the red dotted particles observed by immunofluorescence microscopy represents activated GP IIb/IIIa receptor binding to PDI. (Figure 6E).

## DISCUSSION

Damages to endothelial cell structure and function are important factors in the pathogenesis of vascular diseases [26, 27]. In the diabetic state, chronic hyperglycemia can also cause damage to vascular endothelial cells [28, 29]. MPs are tiny, phospholipid-rich membrane fragments produced during cell germination in response to stimulation or apoptosis [26, 27]. In recent years, many studies have shown that MPs released from damaged endothelial cells play an important role in coagulation [30–32]. Some studies have shown that there is an increase in the level of EMPs in the peripheral circulation of patients with diabetes mellitus, metabolic syndrome, pulmonary arterial hypertension, chronic cardiac failure, or carcinoma [19, 33, 34]. This is a sign of functional impairment of endothelial cells and vascular injury.

Vascular endothelial cadherin, also known as CD144, is a specific marker for endothelial cells [35]. Therefore, CD144-positive MPs were considered EMPs and were isolated. FCM showed that the level of CD144-positive EMPs was the highest in the diabetic group than in the other groups, indicating that endothelial cells were severely damaged in diabetic mice. Moreover, the plasma levels of VWF [36, 37], which reflects damage to vascular endothelium, was found to be significantly higher in the diabetic group than in the other groups. This result indicates the presence of endothelial injury in the blood vessels of diabetic mice.

We increased rutin content for some mice during daily gavage and found that the EMP and EMP-PDI levels in the diabetes plus rutin group were significantly lower than those in the diabetic group, suggesting that rutin has a protective effect on vascular endothelium in the diabetic state and reduces the release of EMP. Jasuja *et al*. [38] have suggested that PDI produced by endothelial cells is the main source of secreted PDI, implying that circulating PDI levels significantly reduced because of the decrease in EMP release in the diabetes plus rutin group.

How does EMP initiate platelet activation? Holbrook *et al.* [22] found that PDI was presented on platelets and endothelial cell-derived particles. PDI is the key enzyme regulating the activation of GP IIb/IIIa receptor to accelerate the thrombosis process. Kim *et al*. [25] have reported that platelet-derived PDI only increased circulating PDI level, which had a cascade amplification effect on platelet activation. Therefore, whether EMP-PDI can initiate early platelet activation in the diabetic state remains unknown. The present study shows that the levels of CD144-positive EMP-PDI were significantly higher in the plasma of the mice in the diabetic group than in that of the mice in the other groups and that platelet activity had significantly increased. The study also found that the expression levels of GP IIb/IIIa receptors and P-selectin, reflecting platelet activation, in normal C57 mice stimulated by EMP in the diabetic group were significantly higher than those in platelets stimulated by EMP in the normal diet group, and the PDI inhibitors RL90 or rutin could effectively reduce the expression of GP IIb/IIIa receptors and P-selectin on platelet surface; this indicates that PDI-induced EMP activation increased in the diabetic state. Thus, EMP-PDI in the plasma of diabetic mice plays an important role in promoting early platelet activation after endothelial cell injury.

Thrombosis requires PDI at every stage. Therefore, the present study focused on whether the use of PDI inhibitors in diabetic mice would lead to changes in platelet activation in the circulation and thus inhibit thrombosis. Bacitracin, a non-specific inhibitor of PDI, is currently the most widely used small-molecule PDI inhibitor [39]. Bacitracin can inhibit proteins and enzymes inside and outside cells, including functional proteins involved in the stability of fibrin [40]. RL90 is an anti-PDI monoclonal antibody, which has a function similar to that of bacitracin [38]. Rutin is another selective inhibitor of PDI [41], which has a structure that prevents it from entering cells. Thus, instead of inhibiting intracellular functional PDI, it only inhibits extracellular PDI.

The platelets of normal C57 mice were stimulated by EMP derived from diabetic mice under different pretreatment conditions. Compared with the EMP-stimulated group, the expression of GP IIb/IIIa and P-selectin on platelets stimulated by EMP pretreated with RL90 or rutin group had significantly decreased, which confirms that RL90 and rutin could effectively reduce platelet activation by inhibiting PDI activity on EMP, thereby inhibiting thrombosis. The effectiveness of rutin suggests that the inhibition of extracellular PDI inhibits platelet activation without affecting intracellular PDI, thus reducing the impact of the treatment on the normal physiological function of cells. This approach, if successfully applied in clinical practice, could substantially reduce the side effects associated with PDI inhibition.

The findings of the present study are consistent with the significant increase in the EMP content in the plasma of diabetic mice because of endothelial cell injury, and we have shown, for the first time, that endothelial PDI plays a key role in early platelet activation in the diabetic state. The PDI inhibitors RL90 and rutin can partially inhibit EMP-PDI and reduce platelet activation, leading to the hypothesis that PDI is a novel target for the treatment of thrombosis.

Further studies should be conducted regarding the mechanism using which rutin can reduce vascular endothelial damage in diabetic patients, which may be another way for rutin to inhibit thrombosis. Moreover, clinical trials should be conducted on the use of rutin for the inhibition of EMP-PDI, and it has to be confirmed whether rutin can also inhibit platelet activation in diabetic patients.

## MATERIALS AND METHODS

### Animal models

Sixty 4-week-old male ApoE^-/-^ mice underwent an intraperitoneal glucose tolerance test (IPGTT) and were then randomly divided into a normal diet group (n = 30) and a high-fat/high-sugar diet group (n = 30). Animals were fed a normal diet or a high-fat/high-sugar diet accordingly for six weeks, followed by an IPGTT. Mice with insulin resistance in the high-fat/high-sugar diet group received an intraperitoneal injection of streptozotocin (STZ) (85–90 mg/kg) (Sigma, USA) for two weeks. Mice with random blood glucose ≥11.1 mmol/L were included in the following experiments as the ApoE^-/-^ mouse model of type 2 diabetes. These mice were divided into the following four groups: normal diet group (n = 15), normal diet plus rutin group (n = 15), diabetes group (n = 15), and diabetes plus rutin group (n = 15). Animals in the normal diet group and the diabetes group received saline (0.1 ml/10 g) daily through gavage, while those in the normal diet plus rutin group and the diabetes plus rutin group received rutin (80 mg/kg) (Tokyo Chemical Industry, Japan) daily through gavage for eight weeks. After terminating treatment, blood samples were collected for subsequent experiments. The mice’s body weight was monitored on a weekly basis. All animal experiments were approved by the Ethics Committee of the Qilu hospital of Shandong University. and all procedures conformed to the guidelines established by Directive 2010/63/EU of the European Parliament regarding the protection of animals used for scientific purposes.

### Isolation of platelets

Blood was collected from the heart apex of each mouse and placed in EP tubes containing 0.109 mol/l sodium citrate at room temperature (RT). The blood samples were centrifuged at 800 rpm/min for 10 mins and platelet-rich plasma was obtained. The platelet-rich plasma was centrifuged at 800 g/min for 10 mins to precipitate platelets. The platelets were re-suspended and their concentration was adjusted to 1 × 106/ml for later use.

### Plasma contents of PDI and von Willebrand factor (VWF) were detected using enzyme-linked immunosorbent assay (ELISA)

The VWF is a glycosylated protein synthesized and secreted by vascular endothelial cells and released into the blood when endothelial cells are damaged [42]. Elevated VWF concentration in blood is a marker of vascular endothelial cell injury. We tested the VWF and PDI contents in mice under different feeding conditions to observe the extent of endothelial cell damage and the corresponding PDI changes. All procedures were performed according to the manufacturer's instructions. The mouse VWF and PDI ELISA kits were purchased from Life Span company.

### Detection of platelet activation markers in mice using flow cytometry (FCM)

P-selectin is a glycoprotein on the platelet membrane. When platelets are activated, P-selectin is exposed to the platelet membrane surface, and its expression on the platelet membrane surface is significantly increased [43]. P-selectin on platelet surface is one of the most specific markers that can reflect platelet activation and thrombosis among the known markers [44]. GP IIb/IIIa is the ultimate pathway of platelet activation. The level of platelet membrane GP IIb/IIIa receptor in plasma can reflect the activity of platelets and is an early marker of platelet activation [10]. Therefore, this study will detect platelet activation by measuring the expression levels of p-selectin and activated GP IIb/IIIa on platelet surface.

The expression of P-selectin on the surface of the isolated platelets was determined. Briefly, 20 μl platelet suspension was placed in a BD FCM test tube, along with 2 μl FITC (Santa Cruz)-labeled, anti-mouse P-selectin antibody and 78 μl phosphate-buffered saline (PBS). After gentle mixing, the mixture was incubated at RT away from light for 20-30 mins. Subsequently, 200 μl 4% paraformaldehyde were added and mixed to fix the platelets, and the samples were immediately subjected to FCM (BD FACS Caliber, BD, USA). To detect GP IIb/IIIa expression on the platelet surface, 20 μl platelet suspension was placed in a BD FCM test tube, along with 5 μl PE-labeled anti-mouse GP IIb/IIIa antibody (JON/A), and 75 μl PBS. After gentle mixing, the mixture was incubated at RT away from light for 30 mins. Finally, 200 μl 4% paraformaldehyde were added. The samples were mixed and immediately subjected to FCM.

### Isolation of EMPs; the presence of PDI in EMPs was detected through CD144-APC and PDI-FITC dual-color FCM, and the plasma level of EMP-PDI in mice was detected

Blood was collected from each mouse’s heart apex and placed in EP tubes containing 0.109 mol/l sodium citrate (1:9) at RT for 10 mins. The blood samples were centrifuged at 300 rpm/min for 15 mins and platelet-poor plasma was obtained. The platelet-poor plasma was centrifuged at 800 g/min for 10 mins and the supernatant was collected. Subsequently, 4 μl CD41-PE and 2 μl CD45-PE were added to the plasma samples and mixed. The mixture was incubated at RT for 30 mins away from light. Then, 20 μl PE-labeled immunomagnetic beads were added and mixed. The mixture was incubated at RT for 20 mins away from light. An LS column was placed in a magnetic field. Magnetic sorting buffer (0.5 ml) was added and allowed to flow through by gravity to prepare the column. The incubated supernatant was loaded onto the sorting column, and the 15-ml centrifuge tube was placed below the column. A magnetic sorting buffer (3 ml) was added to the column and allowed to flow through by gravity. This step was repeated twice. The liquid was centrifuged at 25000 g for 70 mins to obtain the EMP. The magnetic rack for column sorting was manufactured by Miltenyi Biotec (Germany). Subsequently, 2 μl anti-mouse CD144-APC antibody (eBioscience) and 2 μl anti-mouse PDI-FITC antibody (Santa Cruz) were added in a BD tube, followed by 10 μl EMP suspension. PBS was added to a final volume of 100 μl of reaction mixture. The mixture was incubated at 37°C for 20 mins away from light. Finally, 200 μl 4% paraformaldehyde were added. The samples were mixed and immediately subjected to FCM.

### The binding of EMP-PDI to the GP IIb/IIIa receptor on the surface of platelets was confirmed using the Duolink® in-situ proximity ligation assay (PLA)

In-situ PLA detects protein interactions through immunofluorescence. Platelets and EMPs were isolated and processed according to the experimental protocol (i.e., samples were incubated with antibody and PLA probes, followed by ligation and amplification). After mounting, the slides were observed under a fluorescent microscope.

### The platelets of C57 normal mice were stimulated by EMPs with different pretreatment to detect the changes of platelet activity

The anti-PDI monoclonal antibody is RL90, which is a broad spectrum PDI inhibitor [39, 40]. Rutin is a selective inhibitor of PDI [41]. ADP is an agonist for platelet activation [45–47] and was used as a positive control in this study to demonstrate that the incubated stimulated platelets are active and can be activated by agonists to ensure the reliability of the study results.

We pretreated the EMP obtained from each group with ADP, RL90 and rutin, and then stimulated the platelets of healthy C57 mice to observe the effect of pretreated EMPs on the changes of platelet activity in mice. The specific steps are as follows. The EMPs (30 μg/ml) obtained from the different treatment groups were pretreated with adenosine diphosphate (ADP, Chrono-log, USA) (10 μg/ml), RL90 (Abcam) (1 μg/ml) and rutin (60 μM), respectively, at 37 °C for 30 mins. Then, the platelets from healthy C57 mice were stimulated by EMP and different pretreated EMP samples for 30 minutes, respectively. The samples were subjected to FCM for the detection of the expression of platelet activation markers GP IIb/IIIa and P-selectin.

### Statistical analysis

Data are presented as mean ± standard error of the mean, and differences among multiple groups were analyzed using one-way analysis of variance (ANOVA). Statistical graphs were processed using the GraphPad Prism V5.01 software package. Differences were considered statistically significant at *P* < 0.05.
